# Expression of human dCTP pyrophosphatase 1 (DCTPP1) and its association with cisplatin resistance characteristics in ovarian cancer

**DOI:** 10.1111/jcmm.18371

**Published:** 2024-04-30

**Authors:** Yu Wang, Xiangyun Chen, Qiduan Chen, Tiancai Liu, Yingsong Wu, Liping Huang, Yao Chen

**Affiliations:** ^1^ Obstetrics and Gynecology center, Nanfang Hospital Southern Medical University Guangzhou China; ^2^ School of medical laboratory and Biotechnology Southern Medical University Guangzhou China

**Keywords:** cisplatin resistance, cisplatin, DCTPP1, ovarian cancer, ROS

## Abstract

Cisplatin (DDP) resistance is a major challenge in treating ovarian cancer patients. A recently discovered enzyme called dCTP pyrophosphatase 1 (DCTPP1) has been implicated in regulating cancer characteristics, including drug responses. In this study, we aimed to understand the role of DCTPP1 in cancer progression and cisplatin response. Using publicly available databases, we analysed the expression and clinical significance of DCTPP1 in ovarian cancer. Our bioinformatics analysis confirmed that DCTPP1 is significantly overexpressed in ovarian cancer and is closely associated with tumour progression and poor prognosis after cisplatin treatment. We also found that DCTPP1 located in oxidoreductase complex and may be involved in various biological processes related to cisplatin resistance, including pyrimidine nucleotide metabolism, the P53 signalling pathway and cell cycle signalling pathways. We observed higher expression of DCTPP1 in cisplatin‐resistant cells (SKOV3/DDP) and samples compared to their sensitive counterparts. Additionally, we found that DCTPP1 expression was only enhanced in SKOV3/S cells when treated with cisplatin, indicating different expression patterns of DCTPP1 in cisplatin‐sensitive and cisplatin‐resistant cancer cells. Our study further supports the notion that cisplatin induces intracellular reactive oxygen species (ROS) and triggers cancer cell death through excessive oxidative stress. Knocking out DCTPP1 reversed the drug resistance of ovarian cancer cells by enhancing the intracellular antioxidant stress response and accumulating ROS. Based on our research findings, we conclude that DCTPP1 has prognostic value for ovarian cancer patients, and targeting DCTPP1 may be clinically significant in overcoming cisplatin resistance in ovarian cancer.

## 1 INTRODUCTION

Ovarian cancer (OC) is typically diagnosed at an advanced stage, and combination of surgery and chemotherapy is the primary treatment approach. Currently, OC has the lowest 5‐year relative survival rate among all gynaecological malignancies.^1^, ^2^ The low survival rate and high mortality rate of OC are multifactorial, poor chemotherapy response and a high proportion of drug resistance had been considered as the most significant contributors. Related studies suggest that aberrant expression of resistance‐related genes is an important and universal cause of resistance.^3^, ^4^, ^5^, ^6^ Therefore, the mining of drug resistance genes in OC is important to improve the chemotherapy effect.

Nucleotide triphosphate pyrophosphate hydrolase (NTP‐PPase) can hydrolyze the phosphate diester bond of nucleoside triphosphate dNTP to form nucleoside monophosphate dNMP, releasing pyrophosphate.^7^, ^8^, ^9^ The hydrolysis of abnormal nucleotides by NTP‐PPase significantly reduces the number of abnormal nucleotides in the cell nucleotide pool, avoids the incorporation of abnormal nucleotides during DNA synthesis and improves the accuracy of DNA replication.^7^, ^8^, ^10^, ^11^

The DCTPP1 gene is a new member of the NTP‐PPase family and has been predicted as a potential target for antitumour drug development.^12^, ^13^, ^14^, ^15^ DCTPP1 locates on chromosome 16 and encodes a dCTP (deoxycytidine triphosphate) pyrophosphatase.^16^ DCTPP1 gene is highly expressed in the cell nucleus and is involved in maintaining the balance of dCTP levels within the nucleus. The encoded protein primarily functions to hydrolyze excessive dCTP, preventing its excessive accumulation in the cell lead to DNA damage.^16^, ^17^ Studies have demonstrated that DCTPP1 plays a crucial role in regulating nuclear dCTP levels, maintaining DNA stability and resisting DNA damage.^13^, ^16^, ^17^ Additionally, DCTPP1 is also believed to be potentially associated with the development of certain tumours and treatment resistance, although the exact mechanisms remain unclear and require further investigation to elucidate its role in disease.^13^, ^18^, ^19^ Our former study indicated t cisplatin stimulates the overexpression of DCTPP1 in OC cells to stabilize the accumulation of ROS in the cells, thereby protecting the cells from oxidative damage induced by cisplatin.^15^ Currently, the exact role of DCTPP1 in the development, drug metabolism and drug resistance of OC remains unclear.

To gain a comprehensive understanding of its function and its involvement in cisplatin resistance, this study employed bioinformatics methods to link the gene with specific functional categories and biological processes. Furthermore, extensive research was conducted to investigate its role in cisplatin resistance with cisplatin‐sensitive and cisplatin‐resistant cells. The ultimate goal of these findings is to overcome drug resistance and provide novel strategies to enhance the efficacy of chemotherapy in OC.

## 2 METHODS

### 2.1 Prediction the potential functions of DCTPP1

The abnormal expression of DCTPP1 in OC tissue was analysed using the Gene Expression Profiling Interactive Analysis (GEPIA) database. The GEAPIA database loaded relevant data from the TCGA database (https://cancer.nih.gov/) and GTEx (http://commonfund.nih.gov/GTEx/).^20^ Based on the expression data of DCTPP1 gene mRNA in this database, we statistically analysed the expression differences of DCTPP1 gene in normal ovarian tissue and OC tissue. For further exploring the functional interactions between proteins encoded by DCTPP1, functional protein association networks were established by STRING database (https://string‐db.org/).^21^ And Sangerbox^22^ was utilized to perform Gene Ontology (GO) and Kyoto Encyclopedia of Genes and Genomes (KEGG) enrichment analyses of these functional protein association networks. 1‐year survival ROC, 2‐year survival ROC, 3‐year survival ROC and Kaplan–Meier (KM) survival of OC patients were also analysed by Sangerbox tool and KM Plotter database.^23^, ^24^ Furthermore, bioinformatics and text mining performed by Coremine Medical (http://www.coremine.com/medical/#search) was used to confirm whether DCTPP1 associated with OC and drug resistance.^25^, ^26^

### 2.2 Cell culture and DCTPP1 knockdown

Human epithelial ovarian cancer cell lines SKOV3 were purchased from the cell bank of the Chinese Academy of Sciences (Shanghai, China) and cultured in DMEM medium (Gibco, Life Technologies, CA, USA) supplemented with 12% foetal bovine serum (Gibco, Life Technologies, CA, USA) at 37°C in a 5% carbon dioxide humidified environment. The knockdown of the DCTPP1 gene was carried out using a shRNA vector as described earlier.^15^ DCTPP1 knockdown cells established after purinomycin screening were used for subsequent experiments.

### 2.3 Establishment of cisplatin‐resistant cells

To create the cisplatin‐resistant ovarian cancer cells, cisplatin‐sensitive ovarian cancer cells SKOV3 in the logarithmic growth phase, were carefully selected and inoculated into culture dishes. A nominal amount of cisplatin (0.5 μg/mL) was introduced to the medium to facilitate gradual adaptation of the cells to the induced stress. Subsequent to this, the cellular growth was meticulously monitored, while the concentration of cisplatin was systematically augmented to 4 μg/mL over a span of 6 months. Comparative analyses with parental cell lines were conducted to ascertain the drug tolerance capabilities of the resultant drug‐resistant cells. The drug‐resistant cells, named SKOV3/DDP, were frozen in liquid nitrogen for 1 month and then resuscitated and cultured in cisplatin‐free culture medium for 2 months, and its cisplatin IC50 was essentially unchanged. At the same time, the effects of 4 μg/mL cisplatin on the apoptosis of SKOV3 and SKOV3/DDP cells were detected by flow cytometry, which indicated that the cisplatin‐resistant cell line SKOV3/DDP had been successfully established. Subsequent experimental investigations were then carried out on the expanded culture of the drug‐resistant cell lines. To maintain the drug‐resistant phenotype, cisplatin (with final concentration of 2 μg/mL) was added to the culture media for SKOV3/DDP cells.

### 2.4 Immunohistochemical (IHC)

The study and acquisition of patient samples were approved by the Human Ethics Committee of Nanfang Hospital, Southern Medical University (Guangzhou, China). In accordance with the Helsinki Declaration, informed consent was obtained from patients before obtaining primary samples from Nanfang Hospital, Southern Medical University (Guangzhou, China). Immunohistochemical (IHC) detection and determination of results were performed using the IHC method. A wax block containing OC tissue and normal tissue was obtained, followed by incubation with anti DCTPP1 polyclonal antibody (16684‐1‐AP, Proteintech, diluted 1:150) overnight at 4°C. A secondary antibody (goat anti‐mouse IgG HPR) was then added and incubated for 1 h at 25°C. The sections were washed with PBS, underwent DAB colour development, haematoxylin restaining and dehydration using an ethanol and xylene gradient. Finally, the sections were sealed with neutral resin. Images were captured using an ZEISS microscope. Image‐Pro Plus6.0 software (Media Cybernetics, USA) was used to assess the area and density of the dyed region, and the integrated optical density (IOD) value of the IHC section. The mean densitometry of the digital image (magnification, ×200) was designated as representative DCTPP1 staining intensity (indicating the relative DCTPP1 expression level). The signal density of the tissue areas from five randomly selected fields were counted in a blinded manner and subjected to statistical analysis.

### 2.5 Flow cytometry for apoptosis analysis

Annexin V‐APC/7‐AAD Apoptosis Kit and Annexin V‐FITC/PI Apoptosis Kit were purchase from MULTISCIENCES (LIANKE) BIOTECH, CO., LTD, China. Flow cytometry was performed manufacturer's instructions. Cell samples were collected, stained with fluorescent dyes, washed with PBS and analysed using a flow cytometer (BD Pharmingen, USA), and the data generated with at least 10,000 cells counted for each sample was used to determine the apoptotic status of the cells.

### 2.6 Flow cytometry for reactive oxygen species level detection

The dihydroethidium (DHE) staining was utilized to detect the levels of reactive oxygen species (ROS) following the manufacturer's instructions (KeyGEN, China). The procedure was as follows: Cells were harvested and incubated with DHE (20 μM) for 20 min at 37°C in the dark. Relative DHE fluorescence was measured using FACS Calibur flow cytometer (BD Pharmingen, San Diego) based on 5000 gated cell events with Ex/Em = 518 nm/605 nm, and the results were anglicized with Flowjo software.

### 2.7 Fluorescence microscopy for ROS level detection

Each sample was incubated with 20 μM DHE at 37°C for 20 min, while being protected from light. The cells were then washed with PBS, and nuclei were stained with DAPI for 10 min. Finally, DHE fluorescence intensity in cardiac sections was quantified using Image J. Cells were cultured in 24‐well plates at a density of 1 × 10^4^ per well and incubated overnight. Cells were exposed to cisplatin (2 μg/mL, 1 mL) or PBS for 48 h. Subsequently, samples were incubated with 20 μM DHE at 37°C for 20 min and protected from light. Then cells were washed with PBS, and nuclei were stained by DAPI for 10 min. Finally, Image J was used to quantify DHE fluorescence intensity in cardiac section.

### 2.8 CCK‐8 assays

Ovarian cancer cells (1 × 10^3^ cells/well) were plated in 96‐well plates. Following the manufacturer's instructions, the cell counting kit 8 (CCK‐8, Dojindo Molecular Technologies Incorporation, Japan) assay was then employed to quantitate cell death normalized to control conditions according to experiment design. ‘Nonlinear regression (curve fit)’ in Graphpad Prism8.0 was used to calculate the relevant cisplatin IC50 of cells. All experiments were performed at least three times using a minimum of three replicates in each experiment.

### 2.9 RNA extraction and quantitative real‐time PCR

Total RNA was extracted from the cells using Trizol (LEAGENE, China) method. Then, 1 μg of RNA was reverse transcribed into cDNA with the HiFiScript cDNA Synthesis (CWBIO, China). RT‐PCR assay was performed follow the instruction manual of UltraSYBR Mixture (CWBIO, China). RT‐PCR reaction program was set to two steps: (a) preincubation at 95°C for 30 s; (b) 95°C for 5 s, 55°C for 30 s and 72°C for 34 s for 40 cycles. Relative expression was evaluated by the 2^−ΔΔCt^ method with GAPDH as an endogenous control. Primer sequences were listed as follows: DCTPP1 forward: 5′‐TCCATCAGCCTCGGAATCTCCT‐3′, reverse: 5′‐CCTCTTGAAGGGCTGCCCGTT‐3′ and GAPDH forward: 5′‐GAGTCAACGGATTTGGTCGT‐3′, reverse: 5′‐TTGATTTTGGAGGGATCTCG‐3′. Samples were assayed in triplicate using the ABI Prism 7500 detection system (Applied Biosystems, United States).

### 2.10 Western blot

The RIPA buffer (CWBIO, China), containing protease and phosphatase inhibitors, was used for cell sample lysis. The protein concentration of the samples was determined using the BCA assay kit (Beyotime, China). Subsequently, protein samples (30 μg/sample) were separated by sodium dodecyl sulfate‐polyacrylamide gel electrophoresis, transferred onto polyvinylidene fluoride membranes and blocked with 5% skim milk. Commercial antibodies were employed for protein immunoblot analysis, including DCTPP1 (1:1000, 16684‐1‐AP, Proteintech), PARP1 (1:2000, 13371‐1‐AP, Proteintech), Nrf2 (1:1000, AF7623, Beyotime), GCLC (1:1000, AF6969, Beyotime), HO‐1 (1:1000, AF1333, Beyotime) and GAPDH (1:20000, 10494‐1‐AP, Proteintech). The membrane was washed in TBST and subsequently incubated with the relevant secondary antibodies at room temperature for 1 h. The bands were then immersed in the working solution of ECL buffer (Beyotime, CN) for 1 min and exposed in a Chemiluminescence Imaging System with GeneTools software (Syngene, UK). GAPDH was used as the normalized endogenous reference. GAPDH served as an endogenous reference for normalization and the data was homogenized using the control group as the baseline.

### 2.11 Statistical analysis

Statistical analysis was performed using SPSS20.0 and GraphPad Prism 8.0 software (San Diego, CA). Student's *t*‐test or one‐way ANOVA was employed for the analysis. Statistical significance was defined as *p* < 0.05, and all obtained *p* values are presented on the corresponding figures or figure legends, when applicable.

## 3 RESULT

### 3.1 DCTPP1 may be a potential tumour promoter in OC

**FIGURE 1:**
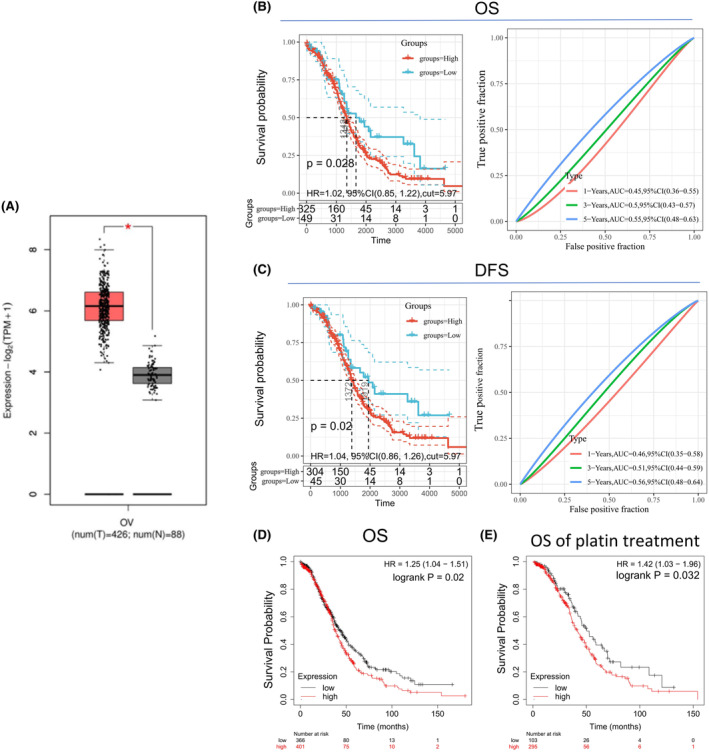
DCTPP1 might be a potential tumour promoter in ovarian cancer. (A) DCTPP1 had significant higher expression level in ovarian cancer specimen compared to normal specimen analysed by GEPIA 2. *p < 0.05. (B, C) Time‐depend ROC curves between the high‐low expression groups of DCTPP1 drawn by Sangerbox online analysis tool, OS (HR = 1.02, *p* = 0.028) and DFS (HR = 1.04, *p* = 0.02). (D, E) Survival curves of OS and OS with cisplatin treatment between the high‐low expression groups of DCTPP1 in KM plotter database, OS (HR = 0.1.25, *p* = 0.02) and OS with cisplatin treatment (HR = 1.51, *p* = 0.0032). OS, overall survival; DFS, disease‐free survival.

To explore the potential role of DCTPP1 in OC, we first analysed the expression of DCTPP1 using a public database. The GEPIA2 online analysis tool found that the DCTPP1 mRNA expression level in OC tissue was significantly higher than that in normal ovarian tissue (*p* < 0.05) (Figure 1A), which is consistent with our previous work.^15^

As shown in Figure 1B,C, time‐dependent ROC curves by Sangerbox online analysis tool show that higher expression levels of DCTPP1 mRNA were significantly correlated with shorter OS (log‐rank *p* = 0.028) and DFS (log‐rank *p* = 0.02). We use the standard metric of Area Under the Curve (AUC) of an ROC curve as the comparison metric, the best AUC of the ROC curve for OS and DFS was 0.55 (95% CI, 0.48–0.63) and 0.56 (95% CI, 0.48–0.63). To further investigated the prognostic potential of DCTPP1 in OC, KM Plotter database was used to evaluate the prognostic value of DCTPP1. As shown in Figure 1D,E, high expression of DCTPP1 was not only associated with worse overall survival but also with the overall survival following cisplatin treatment. The above data suggests that DCTPP1 may be a tumour promoting factor in OC and is associated with the sensitivity of cisplatin chemotherapy for OC.

### 3.2 DCTPP1 might regulate pyrimidine nucleotide metabolism affecting cell growth, division and drug resistance

**FIGURE 2:**
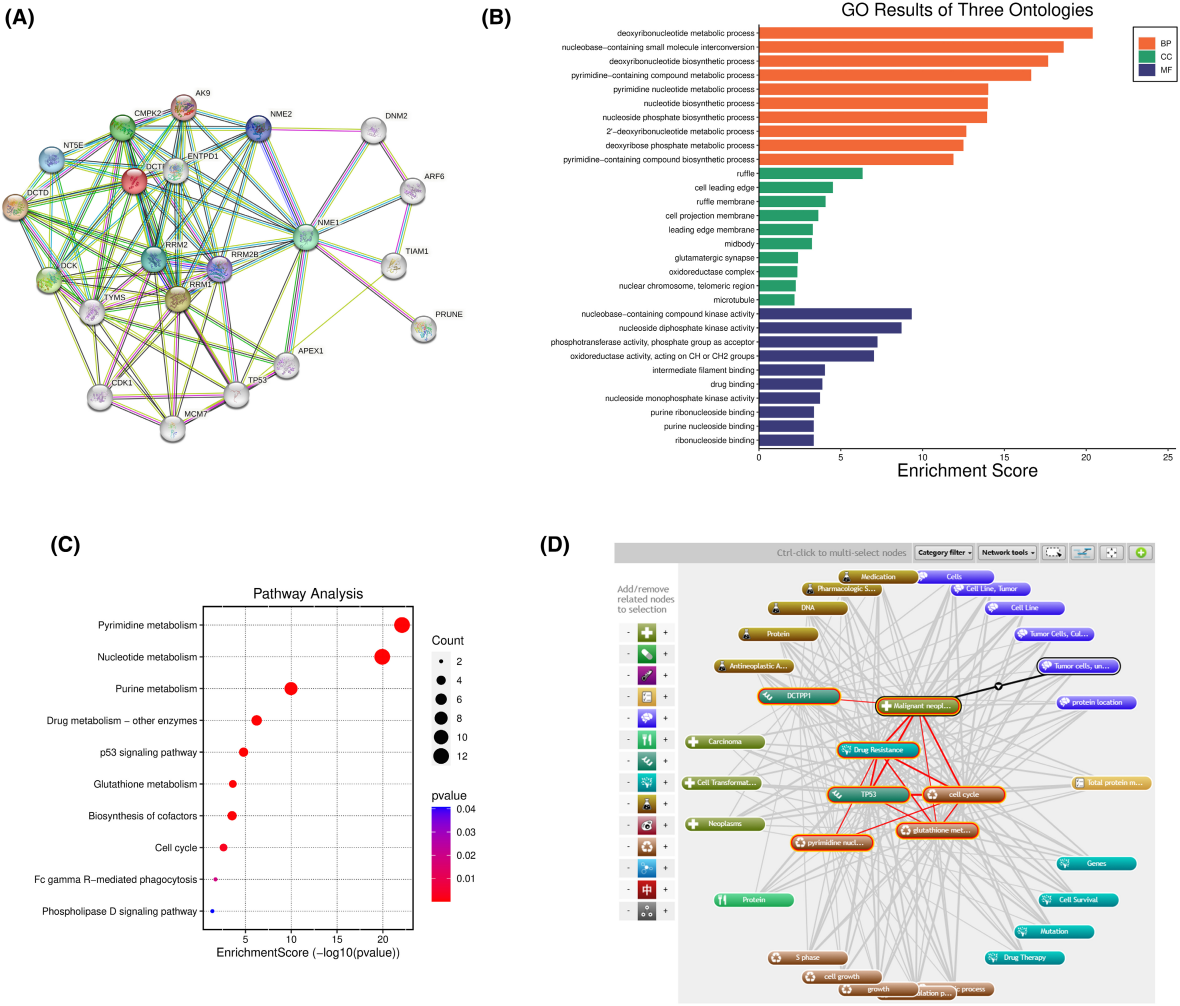
Functional prediction of DCTPP1 gene. (A) The PPI information about CAMK1 evaluated by STRING database. (B, C) GO analysis (B) and KEGG pathway analysis (C) were conducted based on the binding and interaction genes of DCTPP1. (D) Biological processes of dctpp1 mediating drug resistance of ovarian cancer drawn by coremine database.

The function of a gene can be inferred by examining the function of its interacting protein. In this study, the protein–protein interaction (PPI) information for DCTPP1 was assessed using the String database. The results show that a PPI network consists of 21 nodes and 96 edges (PPI enrichment *p* value: <1.0e‐16). Each node represents all proteins produced by a single protein‐coding gene locus, and each edge represents the predicted functional association. DCTPP1 The predicted functional genes mainly include CMPK 2, DCK, NME 1, NME 2, NT5E, RRM 1, RRM 2, RRM 2 B, AK 9, DCTD, TP 53, MCM 7, CDK 1, TYMS, DNM 2, ARF 6, TIAM 1 and PRUNE (Figure 2A). GO analysis of the biological processes, cellular components and molecular functions involved in the above‐mentioned genes revealed that these proteins mainly participate in the metabolism of deoxyribonucleotides, the transformation of small molecules containing nuclear bases, the biosynthesis of deoxyribonucleotides, the metabolism of pyrimidine complexes, the biosynthesis of nucleoside phosphate and the metabolism of pyrimidine nucleoside. The genes associated with DCTPP1 are located in various cellular compartments, including cell membranes, telomere regions of nuclear chromosomes, cell folds, microtubules, oxidoreductase complexes and glutamatergic synapses. These genes exhibit molecular functions such as complex kinase activity involving nucleoside diphosphate kinase activity, phosphotransferase activity, oxidoreductase activity, intermediate filament binding nucleoside monophosphate kinase activity and purine ribonucleoside binding (Figure 2B). The results of KEGG pathway enrichment analysis indicate that the DCTPP1 interacting proteins are significantly enriched in various pathways, including pyrimidine metabolism, purine metabolism, drug metabolism, other enzyme p53 signalling pathways, glutathione metabolism, cell cycle, iron γ R‐mediated phagocytosis and phosphatase D signalling pathway (Figure 2C). Most of these signalling pathways are associated with drug resistance in OC (Figure 2D). The above results indicate that DCTPP1 may act on drug metabolism, cell cycle and various biological functions of OC cells by affecting nucleotide metabolism.

### 3.3 DCTPP1 shows disparities in the expression levels and regulatory patterns in cells with varying sensitivity to cisplatin

**FIGURE 3:**
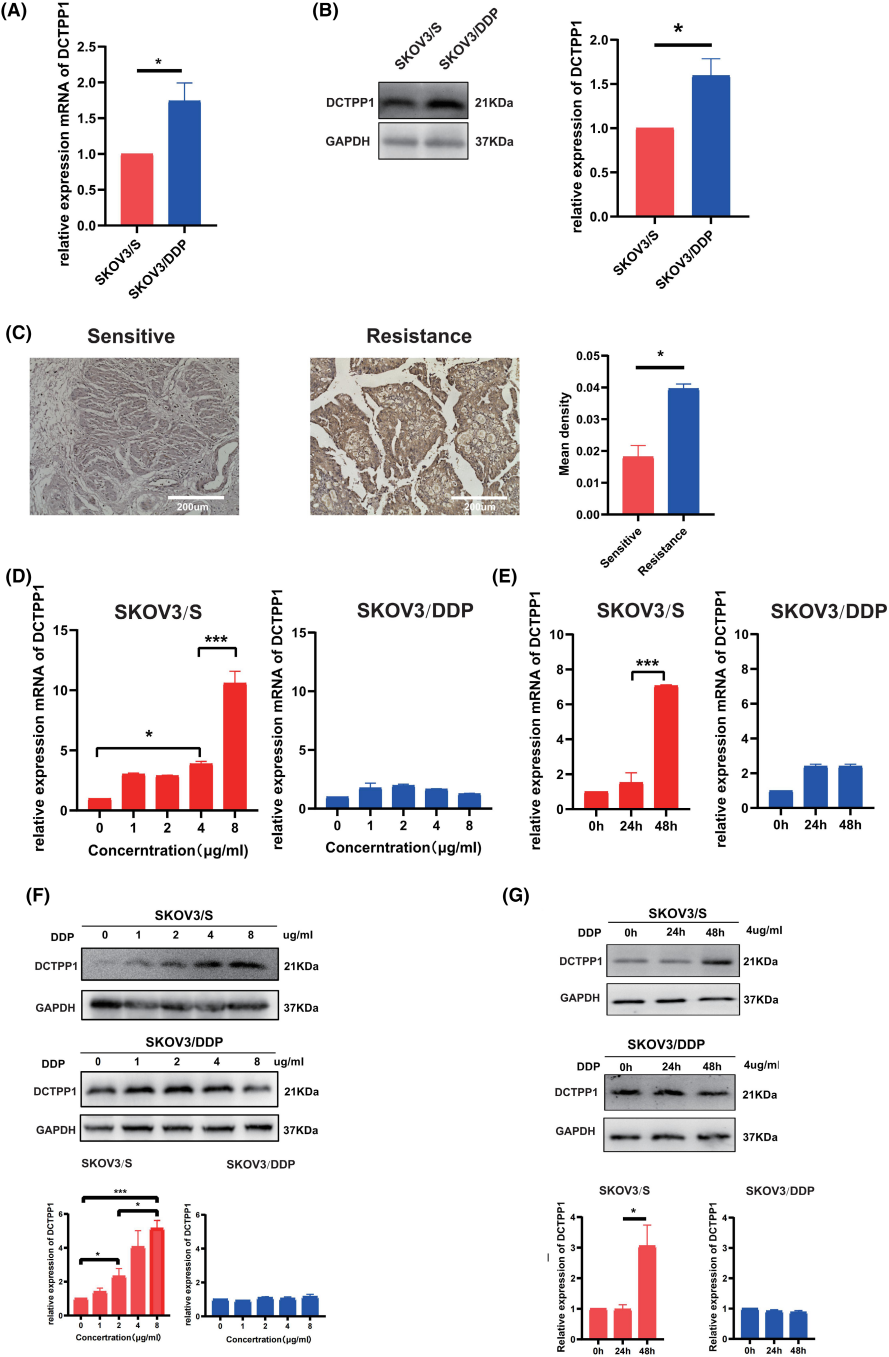
DCTPP1 shows disparities in the expression levels and regulatory patterns in cells with varying sensitivity to cisplatin. (A) Detection of DCTPP1 expression in SKOV3/S and SKOV3/DDP cells with real‐time q‐PCR. (B) Detection of DCTPP1 expression in SKOV3/S and SKOV3/DDP cells with Western blot. (C) Immunohistochemistry staining of DCTPP1 or platinum sensitivity and resistance in patient samples (Scar bar = 200 μm). (D) Detection of DCTPP1 expression in SKOV3/S and SKOV3/DDP cells treated with cisplatin at different concentrations with real‐time q‐PCR. (E) Detection of DCTPP1 expression in SKOV3/S and SKOV3/DDP cells treated with cisplatin (4 μg/mL) at different timepoint with real‐time q‐PCR. (F) Detection of DCTPP1 expression in SKOV3/S and SKOV3/DDP cells treated with cisplatin at different concentrations with western blot. (G) Detection of DCTPP1 expression in SKOV3/S and SKOV3/DDP cells treated with cisplatin (4 μg/mL) at different timepoint with Western blot. The results are presented as mean ± SEM (*p ≤ 0.05, ***p ≤ 0.001).

Cisplatin is the preferred drug for OC. In order to clarify the relationship between DCTPP1 expression and cisplatin sensitivity, this study established cisplatin resistant SKOV3/DDP cells based on cisplatin sensitive SKOV3/S through gradient induction (Figure S1), and detected the basic expression level of DCTPP1 in SKOV3/S cells and SKOV3/DDP cells. The results demonstrated a significant higher expression level of DCTPP1 in SKOV3/DDP cells compared to SKOV3/S cells, both at the mRNA and protein levels (Figure 3A,B). The experimental results from IHC indicate that the expression of DCTPP1 is significantly elevated in cisplatin‐resistant clinical samples compared to cisplatin‐sensitive clinical samples (Figure 3C). To investigate whether the expression of DCTPP1 is influenced by cisplatin, we examined the expression of DCTPP1 with various concentrations of cisplatin (0–8 μg/mL) for 48 h or treated with cisplatin (4 μg/mL) for 24 and 48 h. Our findings revealed an enhancement of DCTPP1 expression in cisplatin‐treated SKOV3/S cells with time‐dependent and dose‐dependent manner in mRNA level, while the changes in DCTPP1 expression levels were relatively insignificant in SKOV3/DDP cells (Figure 3D,E). Immunoblotting also showed consistent results at the protein level (Figure 3F,G). These results suggest that there are disparities in the expression levels and regulatory patterns of DCTPP1 in cells with varying sensitivity to cisplatin, indicating its potential involvement in cisplatin resistance in OC.

### 3.4 SKOV3/DDP counteract the cytotoxic effects of cisplatin by attenuating excessive ROS production

**FIGURE 4:**
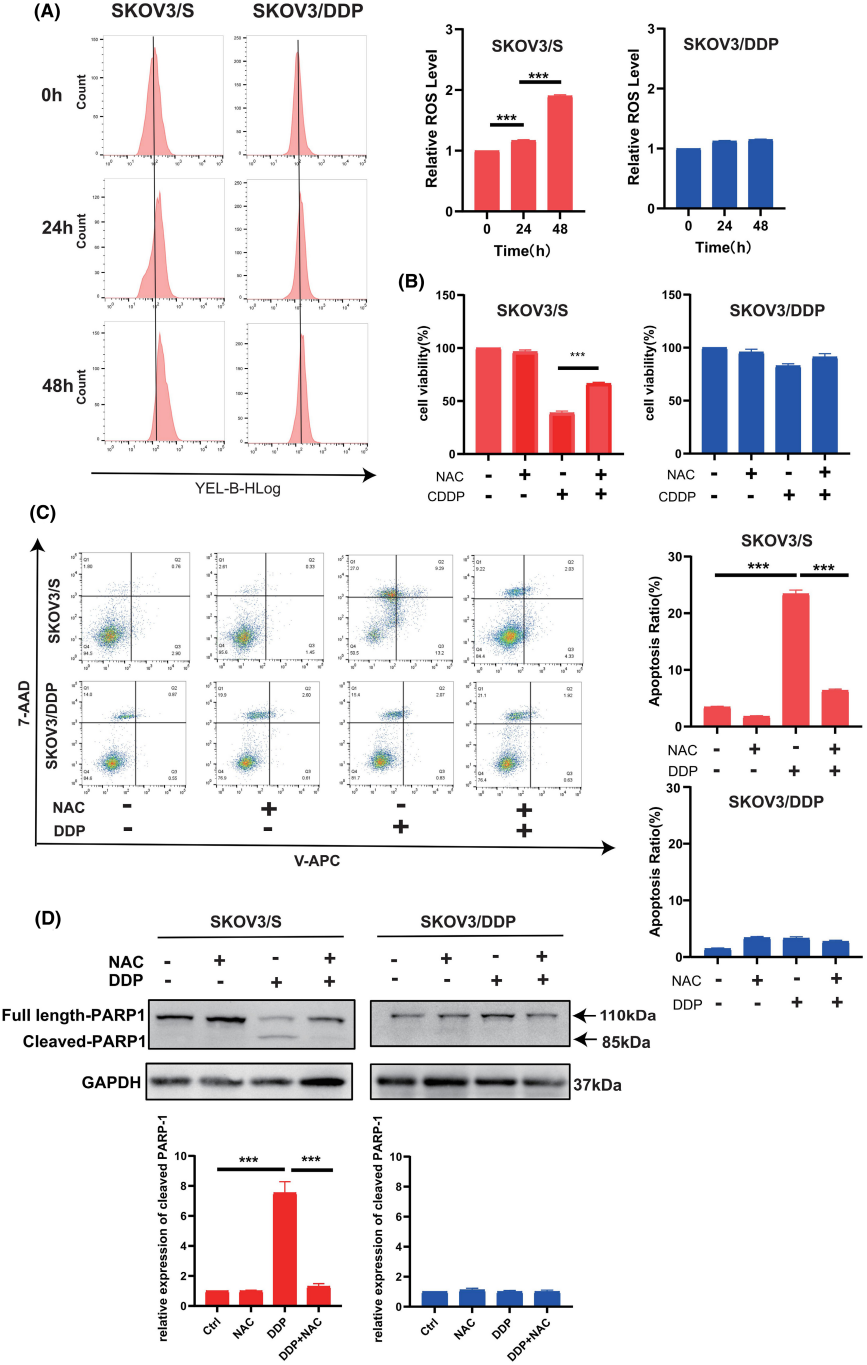
SKOV3/DDP counteract the cytotoxic effects of cisplatin by attenuating excessive ROS production. (A) The intracellular reactive oxygen species (ROS) levels of the SKOV3/S and SKOV3/DDP cells treated with cisplatin (4 μg/mL) for 24 and 48 h. ROS were measured by flow cytometric assay using dihydroethidium (DHE) staining. (B) Cell viability of the SKOV3/S and SKOV3/DDP cells treated with cisplatin (4 μg/mL) only or cotreated with NAC. (C) Cell apoptosis of the SKOV3/S and SKOV3/DDP cells treated with cisplatin 4 μg/mL only or cotreated with NAC. (D) Apoptosis‐related protein PARP‐1 expression of SKOV3/S and SKOV3/DDP cells. SKOV3/S and SKOV3/DDP cells treated with cisplatin (4 μg/mL) only or cotreated with NAC (right). The results are presented as mean ± SEM (*p ≤ 0.05, ***p ≤ 0.001).

The cytotoxicity of cisplatin in OC cells is associated with cisplatin‐induced ROS accumulation. In GO analysis, we found that DCTPP1 aggregates in the oxidoreductase complex. Our previous work has shown that knockdown of DCTPP1 leads to excessive production of ROS in OC cells, increasing their sensitivity to cisplatin treatment.^15^ Therefore, in this study, we examined the accumulation of ROS in SKOV3/S cells and SKOV3/DDP cells after cisplatin treatment. The results showed that the levels of ROS significantly increased in SKOV3/S cells after 24 h and 48 h of cisplatin treatment, while the changes in ROS levels in SKOV3/DDP cells were not significant (Figure 4A). Subsequent investigations demonstrated that the coadministration of cisplatin with N‐acetyl‐l‐cysteine (NAC), a scavenger of reactive oxygen species (ROS), resulted in a significant inhibition of cisplatin‐induced cell death in SKOV3/S cells. However, no significant changes were observed in SKOV3/DDP cells, as assessed by CCK‐8 assay and flow cytometry. (Figure 4B,C). The same phenomenon can also be observed in the expression of apoptosis‐related protein PARP‐1 (Figure 4D).

These results suggest a connection between DCTPP1 and cisplatin resistance in OC, which is mediated through oxidative stress. The findings lead us to speculate that the expression of DCTPP1 may be involved in cisplatin resistance by assisting OC cells in coping with cisplatin‐induced oxidative stress.

### 3.5 Knockdown DCTPP1 could restore SKOV3/DDP to cisplatin‐induced ROS overload

**FIGURE 5:**
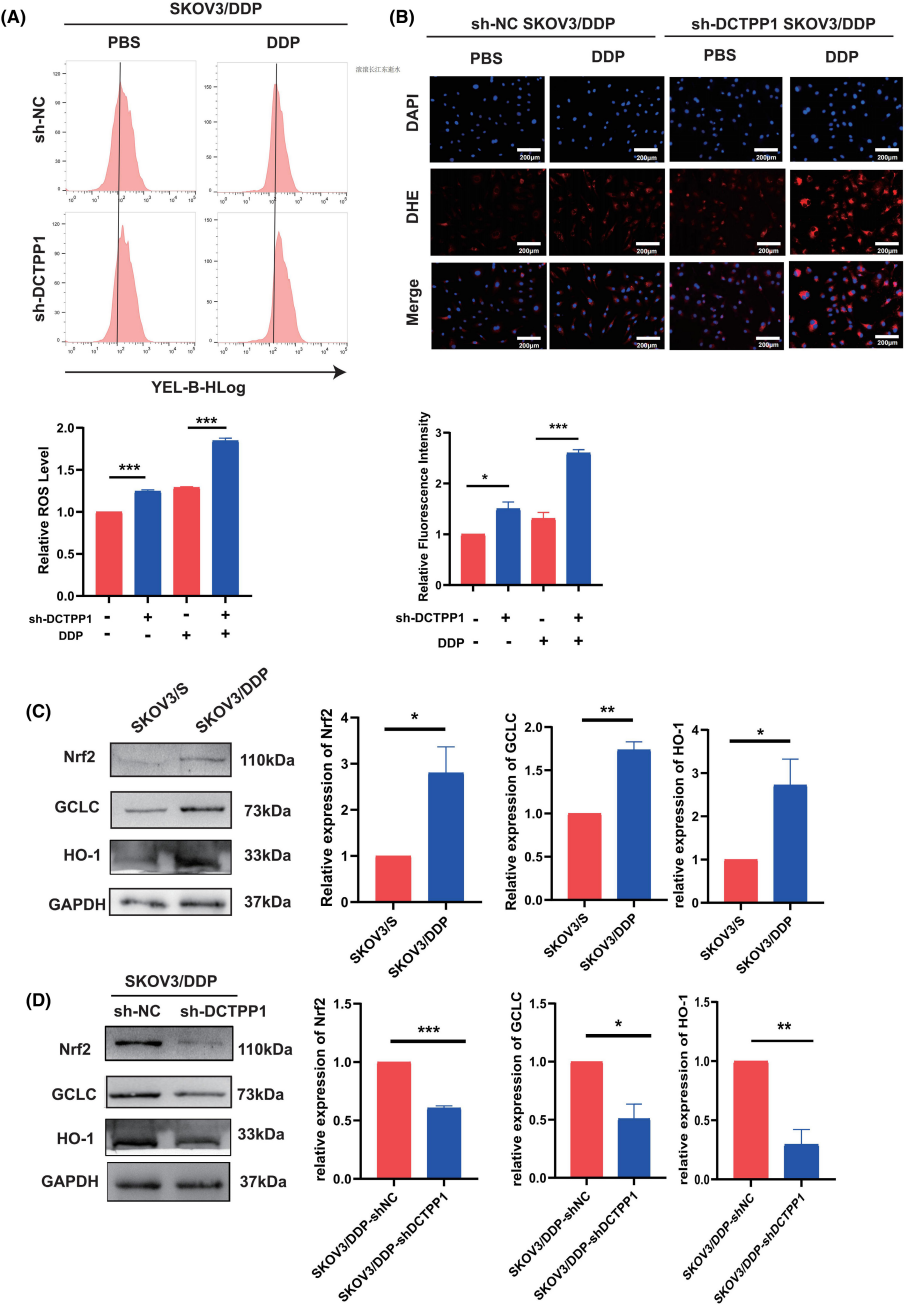
Knockdown DCTPP1 could restore SKOV3/DDP to cisplatin‐induced ROS overload. (A) The intracellular reactive oxygen species (ROS) levels of the SKOV3/DDP cells treated with cisplatin (4 μg/mL) for 24 h. The cells were divided into shNC group and shDCTPP1 group. ROS were measured by flow cytometric assay using dihydroethidium (DHE) staining. (B) DHE staining results were recorded using fluorescence microscope (Scar bar = 200 μm). (C) Immunoblotting analysis of antioxidant related proteins HO‐1, Nfr2 and GCLC of SKOV3/S and SKOV3/DDP. (D) Immunoblotting analysis of antioxidant related proteins HO‐1, Nfr2 and GCLC of SKOV3/DDP. The cells were divided into shNC group and shDCTPP1 group. The results are presented as mean ± SEM (*p ≤ 0.05, **p ≤ 0.01, ***p ≤ 0.001).

The endogenous expression of DCTPP1 was suppressed using lentiviral shRNA targeting DCTPP1 (Figure S2) in order to elucidate the potential involvement of DCTPP1 in the development of cisplatin resistance in OC, specifically by modulating intracellular reactive ROS levels. According to the findings depicted in Figure 5A,B, the utilization of flow cytometry and immunofluorescence methodologies revealed a notable augmentation in intracellular reactive ROS levels subsequent to the knockout of the DCTPP1 gene in comparison to the control cells. Previous research has indicated that the Nrf2/HO‐1 signalling pathway plays a crucial role in modulating intracellular oxidative stress homeostasis, both in the presence and absence of cisplatin treatment, and it is also believed to limit intracellular ROS accumulation.^15^, ^27^ Our previous investigations have provided evidence that the knockdown of DCTPP1 leads to an excessive accumulation of reactive ROS in OC cells, primarily by exerting a negative regulatory effect on the Nrf2/HO‐1 signalling pathway.^15^ Then, we compared the expression levels of Nrf2 and HO‐1 between SKOV3/S cells and SKOV3/DDP cells. Our results, as depicted in Figure 5C, demonstrated that both mRNA and protein levels of Nrf2 and HO‐1 were significantly higher in SKOV3/DDP cells compared to SKOV3/S cells. Subsequently, we examined the impact of DCTPP1 knockdown on the expression of Nrf2 and HO‐1. Notably, our findings, as illustrated in Figure 5D, revealed a significant reduction in the expression levels of Nrf2 and HO‐1 in SKOV3/DDP cells with DCTPP1 knockdown, as compared to the parental cells. Collectively, these results substantiate our hypothesis that the knockdown of DCTPP1 induces an overwhelming accumulation of ROS in drug‐resistant OC.

### 3.6 Reversal of cisplatin resistance in SKOV3/DDP cells through DCTPP1 knockdown

**FIGURE 6:**
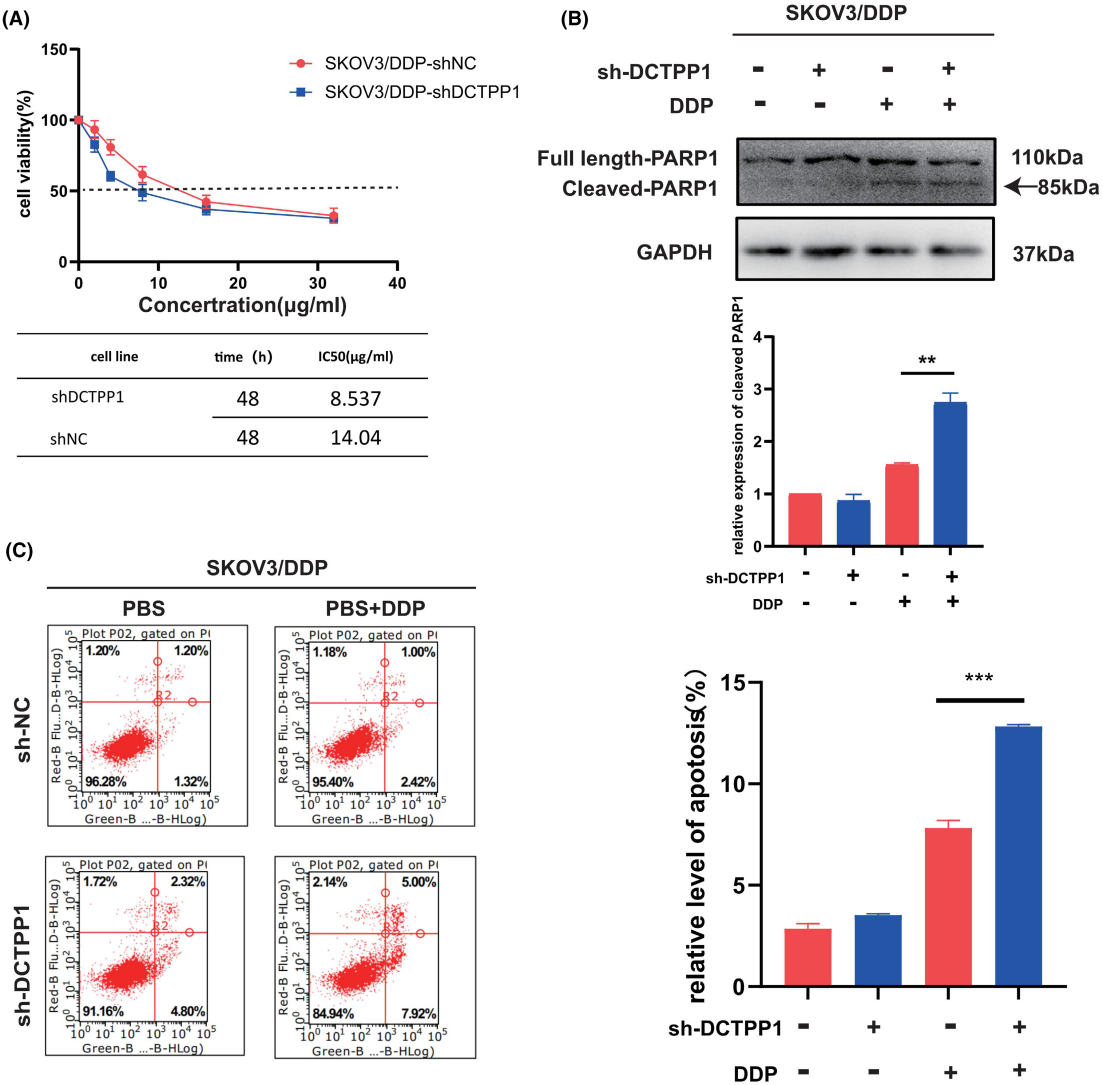
Reversal of cisplatin resistance in SKOV3/DDP cells through DCTPP1 knockdown. (A) The IC50 values of cisplatin for SKOV3/DDP cells. The cells were divided into shNC group and shDCTPP1 group. (B) Apoptosis‐related protein PARP‐1 expression of SKOV3/DDP cells transfected with shNC and shDCTPP1. SKOV3/DDP cells treated with cisplatin (4 μg/mL) or PBS. (C) Apoptosis ratio of SKOV3/DDP cells transfected with shNC and shDCTPP1. SKOV3/DDP cells treated with cisplatin (4 μg/mL) or PBS. The results are presented as mean ± SEM (**p ≤ 0.01, ***p ≤ 0.001).

The potential of DCTPP1 knockdown in reversing the drug resistance phenotype of OC cells through ROS overload was further explored. Firstly, the cell viability and cisplatin IC50 values were compared between parental SKOV3/DDP cells and DCTPP1 knockdown cells using the CCK8 assay. The results showed a significant decrease in the cisplatin IC50 in the DCTPP1 knockdown group compared to the parental control group (Figure 6A). Subsequently, the impact of DCTPP1 knockdown on cisplatin‐induced apoptosis in OC cells was evaluated. It was observed that DCTPP1 knockdown significantly increased the apoptosis rate of SKOV3/DDP cells in the presence of cisplatin (Figure 6B). Additionally, enhanced cleavage of PARP‐1, a marker of apoptosis, was observed in DCTPP1 knockdown cells treated with cisplatin (Figure 6C).

These findings suggest that the resistance can be reversed through the knockdown of DCTPP1, leading to increased apoptosis and sensitization to cisplatin treatment.

## 4 DISCUSSION

Gene function analysis is crucial for identifying new targets and improving disease diagnosis and treatment.^22^, ^28^, ^29^ In this study, the results of bioinformatics mining not only demonstrate the high expression of DCTPP1 in ovarian cancer and its correlation with disease prognosis, but also suggest its principal biological function to be centred around nucleotide metabolism, particularly in the regulation of pyrimidine metabolism, involving the P53 signalling pathway, cell cycle and drug metabolism. The results highlighted DCTPP1's involvement in nucleotide metabolism, consistent with its known enzymatic activity as a dCTP pyrophosphatase. Dysregulation of pyrimidine nucleotide metabolism is implicated in cancer progression, making it a relevant area of study in OC. In tumour cells, chemical agents and the abnormal nucleotide metabolism resulting from rapid proliferation often lead to the generation of a large quantity of intermediates and abnormal nucleotides, triggering the activation of the P53 signalling pathway, thereby inducing cell apoptosis or cell cycle arrest to prevent further proliferation of damaged cells.^30^, ^31^, ^32^, ^33^ Tumours require certain mechanisms to regulate the balance of pyrimidine metabolism in response to the pressure of rapid proliferation and the effects of chemotherapeutic agents.^28^, ^29^, ^34^ Studies have indicated that DCTPP1 exhibits a strong preference for abnormal dCTP, with the highest activity observed for 5‐iodo‐dCTP, followed by 5‐bromo‐dCTP, unmodified CTP, 5‐methyl‐dCTP and 5‐chloro‐dCTP. This may serve to protect DNA or RNA from the insertion of noncanonical nucleoside triphosphates.^11^, ^12^ These findings prompt further investigation into the potential involvement of DCTPP1, as a nucleotide balance enzyme, in cisplatin resistance in ovarian cancer.

To investigate the role of DCTPP1 in OC cell resistance, we established cisplatin‐resistant SKOV3/DDP cells. Interestingly, the expression levels of DCTPP1 were found to be significantly lower in cisplatin‐sensitive OC cells compared to their resistant counterparts, and the regulatory patterns of DCTPP1 in cisplatin‐sensitive and drug‐resistant cells exhibit notable distinctions. Moreover, consistent with these findings, clinical specimens exhibiting clinical manifestations of cisplatin resistance displayed higher expression of DCTPP1 compared to specimens demonstrating cisplatin sensitivity. These findings suggest that DCTPP1 may be involved in cisplatin resistance in OC.

A significant portion of the cytotoxicity of cisplatin arises from its ability to induce nucleotide mispairing and disrupt cellular redox balance.^35^, ^36^ However, resistant cells exhibit enhanced ability to cope with oxidative stress, resulting in platinum resistance.^37^, ^38^ DCTPP1, as a dCTP pyrophosphatase, is believed to play a role in inhibiting the excessive accumulation of ROS in tumour cells by clearing aberrant nucleotides. In previous studies,^16^ our former findings demonstrate that the knockdown of DCTPP1 leads to an excessive accumulation of ROS in OC cells, primarily by exerting a negative regulatory effect on the Nrf2/HO‐1 signalling pathway.^15^ In this study, the investigation of intracellular oxidants has provided compelling evidence supporting the notion that cisplatin‐resistant cells employ a mechanism to mitigate the cytotoxic effects of cisplatin by dampening the overproduction of reactive ROS.^39^ Moreover, our study reveals that the expression levels of Nrf2 and HO‐1 are significantly higher in cisplatin‐resistant cells compared to cisplatin‐sensitive cells, further supporting the role of these factors in mediating cisplatin resistance. The Nrf2/HO‐1 signalling pathway plays a pivotal role in mediating cisplatin resistance in OC cells. Nrf2, a transcription factor, acts as a central regulator of cellular antioxidant defence mechanisms.^40^ Upon activation, Nrf2 induces the expression of key antioxidant and detoxification enzymes, including HO‐1 and GCLC, which serve as downstream effectors of the Nrf2 pathway. HO‐1 and GCLC play critical roles in cellular defence against oxidative stress by promoting the degradation of heme and the synthesis of glutathione, respectively.^27^, ^41^, ^42^ Importantly, we demonstrated that downregulation of DCTPP1 can reverse cisplatin resistance, thereby increasing cellular apoptosis and sensitivity to cisplatin treatment. This was accompanied by an accumulation of intracellular reactive oxygen species and inhibition of the antioxidant pathway NFR2/HO‐1.

While this study provides valuable insights into the role of DCTPP1 in ovarian cancer (OC), there are still some limitations. Firstly, the study is primarily based on in vitro experiments, and therefore, further in vivo studies are required to confirm the impact of DCTPP1 on cisplatin resistance, in order to gain a more comprehensive understanding of the role of DCTPP1 in OC treatment. Additionally, this study only utilized shRNA to achieve DCTPP1 knockdown, primarily analysing the role of DCTPP1 based on changes in gene expression levels. The screening and use of inhibitors targeting the enzymatic activity of DCTPP1 will enable a more in‐depth exploration of its role in cisplatin resistance in ovarian cancer (OC) and the potential of it serving as a cisplatin treatment enhancer. This will contribute to a more comprehensive understanding of the role of DCTPP1 in OC treatment and provide valuable insights for future clinical applications.

## 5 CONCLUSION

In conclusion, our study highlights the crucial role of DCTPP1 in cisplatin resistance in OC. DCTPP1 may impact cell growth, division and drug resistance in OC by regulating pyrimidine nucleotide metabolism. Knockdown of DCTPP1 in cisplatin‐resistant cells disrupts their ability to tolerate oxidative stress and restores their sensitivity to cisplatin. Our findings provide new insights for overcoming cisplatin resistance in OC, and further investigations are warranted to elucidate the underlying mechanisms.

## AUTHOR CONTRIBUTIONS

**Yu Wang:** Investigation (equal); writing – original draft (equal). **Xiangyun Chen:** Investigation (equal); writing – original draft (equal). **Qiduan Chen:** Investigation (supporting); visualization (equal). **Tiancai Liu:** Data curation (equal); software (equal). **Yingsong Wu:** Funding acquisition (supporting); resources (equal). **Liping Huang:** Project administration (equal); writing – review and editing (equal). **Yao Chen:** Project administration (equal).

## CONFLICT OF INTEREST STATEMENT

The authors declare no competing interests.

## Supporting information


Figure S1.

Figure S2.

## Data Availability

Data available on request from the authors.
