# Supplementary material for: Expression of human dCTP pyrophosphatase 1 (DCTPP1) and its association with cisplatin resistance characteristics in ovarian cancer
Source: J Cell Mol Med. 2024 Apr 30;28(9):e18371. doi: 10.1111/jcmm.18371 (PMC11058668; doi:10.1111/jcmm.18371)
Supplement: Supplementary file 1 — Figure S1. [file JCMM-28-e18371-s001.zip › FigureS1 caption.docx]

Figure S1. A cisplatin-resistant cell line, SKOV3/DDP, was developed by subjecting SKOV3 cells to prolonged exposure to cisplatin. (A) SKOV3/S and SKOV3/DDP cells were exposed to various concentrations of cisplatin for 24 and 48 hours. Cell viability was assessed using the CCK-8 assay, and the resulting data was used to generate best-fit curves to determine the IC50 values for SKOV3/S and SKOV3/DDP cells. (B) SKOV3/S and SKOV3/DDP cells were treated with varying concentrations of cisplatin (0 to 16 µg/mL) for 48 hours. Cell apoptosis was assessed by flow cytometry using Annexin V staining. (C) The expression levels of apoptosis-related proteins were analyzed through Western blotting. (D) SKOV3/S and SKOV3/DDP cells were treated with cisplatin (4 µg/mL) for 24 and 48 hours. Cell apoptosis was quantified using flow cytometry with Annexin V staining.The presented results are expressed as mean ± standard error of the mean (SEM) (* p ≤ 0.05, ** p ≤ 0.01).
