# Supplementary figures and images for: Expression of human dCTP pyrophosphatase 1 (DCTPP1) and its association with cisplatin resistance characteristics in ovarian cancer
Source: J Cell Mol Med. 2024 Apr 30;28(9):e18371. doi: 10.1111/jcmm.18371 (PMC11058668; doi:10.1111/jcmm.18371)

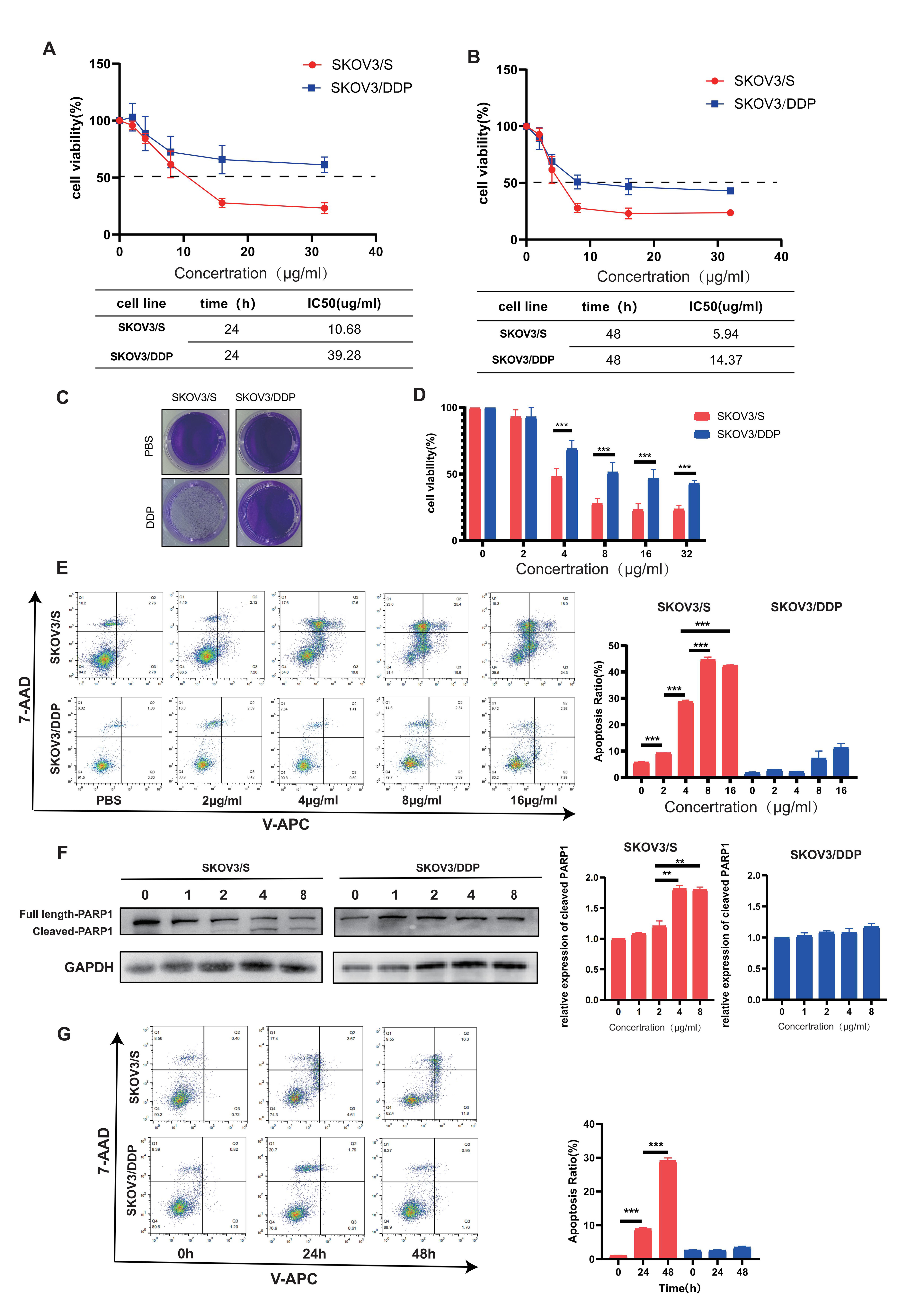

Supplement: Supplementary file 1 — Figure S1. [file JCMM-28-e18371-s001.zip › Figure S1.jpg]

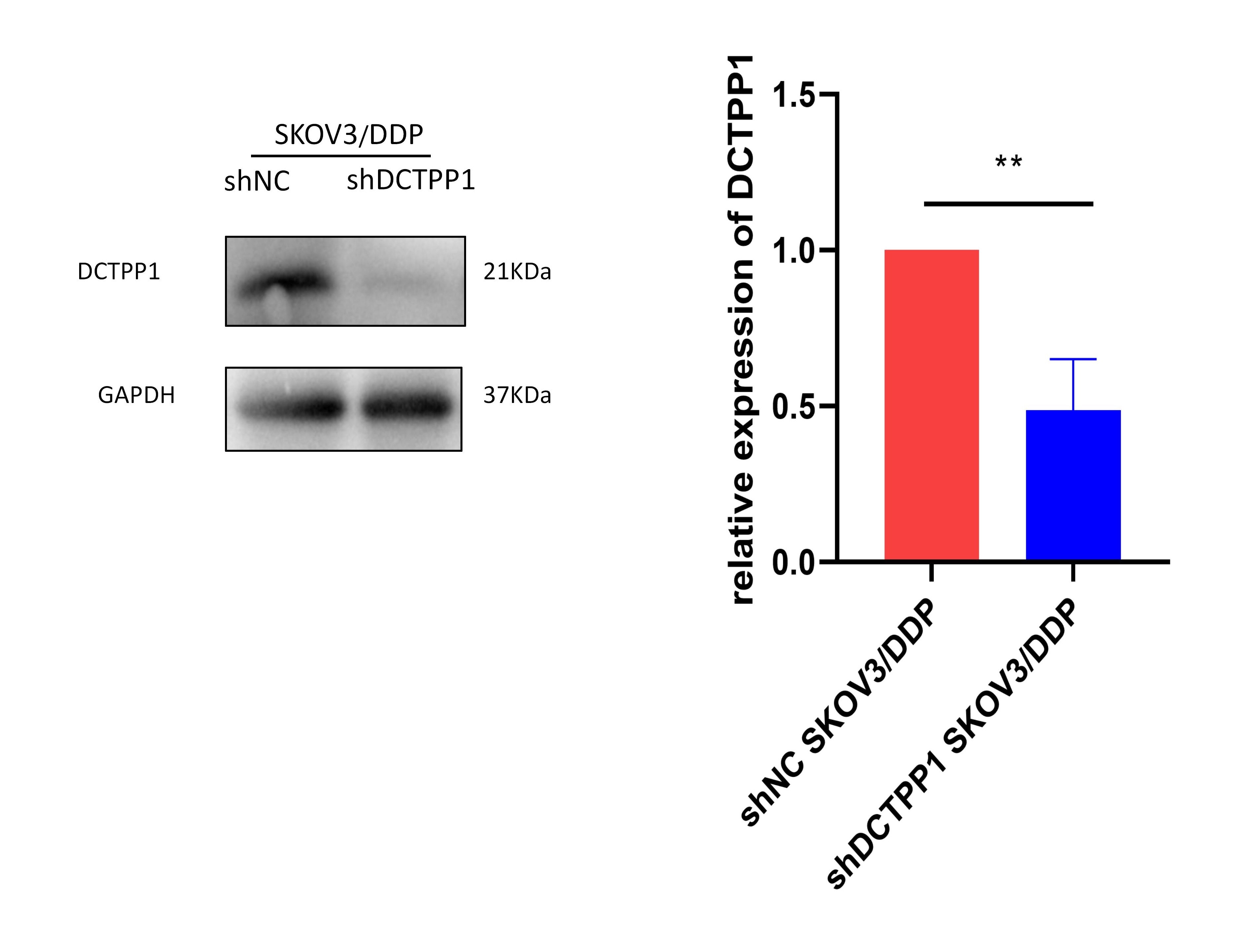

Supplement: Supplementary file 2 — Figure S2. [file JCMM-28-e18371-s002.zip › Figure S2.jpg]
