# Supplementary material for: Expression of human dCTP pyrophosphatase 1 (DCTPP1) and its association with cisplatin resistance characteristics in ovarian cancer
Source: J Cell Mol Med. 2024 Apr 30;28(9):e18371. doi: 10.1111/jcmm.18371 (PMC11058668; doi:10.1111/jcmm.18371)
Supplement: Supplementary file 2 — Figure S2. [file JCMM-28-e18371-s002.zip › FigureS2 caption.docx]

FigureS2. Knockdown of DCTPP1 in SKOV3/DDP cells. SKOV3/DDP cells were transfected with shRNA against DCTPP1 plasmid or negative control plasmid .The expression levels of DCTPP1 proteins were analyzed through Western blotting. The presented results are expressed as mean ± standard error of the mean (SEM) (* p ≤ 0.05, ** p ≤ 0.01).
